# The Diseases of the Nose, Throat and Ear

**Published:** 1907-03

**Authors:** 


**The Diseases of the Nose, Throat and Ear.** By **Charles P. Grayson,** M.D., Clinical Professor of Laryngology, Medical Department, University of Pennsylvania. Second edition, revised and enlarged. Octavo, about 550 pages, with 152 engravings and 15 plates in black and colors. Philadelphia and New York: Lea Brothers & Co. 1906. (Cloth, $4.00, net).

The most salient feature of the first edition of this book was the selection by the author of just what his readers wanted to know and the very delightful and understandable manner of presentation.

It is easy to overdo in dealing with a specialty and write a bulky volume, heavy-laden with verbosity; it requires the work of a literary genius as well as a master of the field, to present an entire subject in so compact a volume, so intelligent a fashion.

Not only does the author tell what to do in any case under consideration but he tells how to do it and removes all doubt by adding the why. Each condition is taken up in detail and the best therapeutic treatment is outlined. There is much new material in the text and many new illustrations. It is not necessarily a book which will appeal only to the specialist; it has a personal interest for the general practitioner.

